# Multiple Mental Foramina: A Rare Anatomical Variation Detected by Cone-Beam Computed Tomography (CBCT)

**DOI:** 10.7759/cureus.63524

**Published:** 2024-06-30

**Authors:** Maryam Mohebiniya, Mobina Kamani

**Affiliations:** 1 Department of Oral and Maxillofacial Radiology, School of Dentistry, Arak University of Medical Sciences, Arak, IRN; 2 Department of Periodontics, School of Dentistry, Arak University of Medical Sciences, Arak, IRN

**Keywords:** accessory mental foramen, mandible, cone-beam computed tomography, anatomic variation, mental foramina

## Abstract

The mental foramen is a single anatomical structure that can be seen bilaterally in the body of the mandible and generally in the lower area of the premolars. Sometimes, the mental foramen can have accessory foramina that should be considered. Clinical evaluation of the accessory mental foramina is critical because of its neurovascular fibers. Identifying the secondary mental foramen reduces the possibility of paraesthesia and pain after surgery.

## Introduction

The mental foramen is the anterior limit of the mandibular canal. There are different anatomical variations in its position; it might be seen in the area between the mesial first premolar of the mandible and the mesial first molar of the mandible. However, it is usually seen in the area of the apex of the second premolar of the mandible and the middle half between the lower cortex of the mandible and the alveolar ridge crest [1,2]. Assessing the anatomical position of the mental foramen is very important in implant placement and surgical treatment planning, and special attention should be paid to its position and the presence of the secondary mental foramen [3,4].

The present case report involves a patient with a particular anatomical variation in the mental foramen position detected by cone-beam computed tomography (CBCT).

## Case presentation

**Figure 1:**
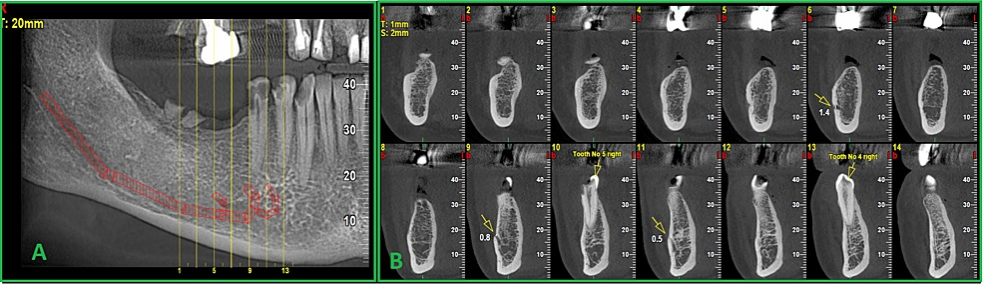
A) Reformatted panoramic view and B) serial cross-sectional view showing triple mental foramina In panel B, the three openings of the mandibular canal as multiple mental foramina are shown in section numbers 6, 9, and 11. In each section, the diameter of the foramen is written in millimeters.

**Figure 2:**
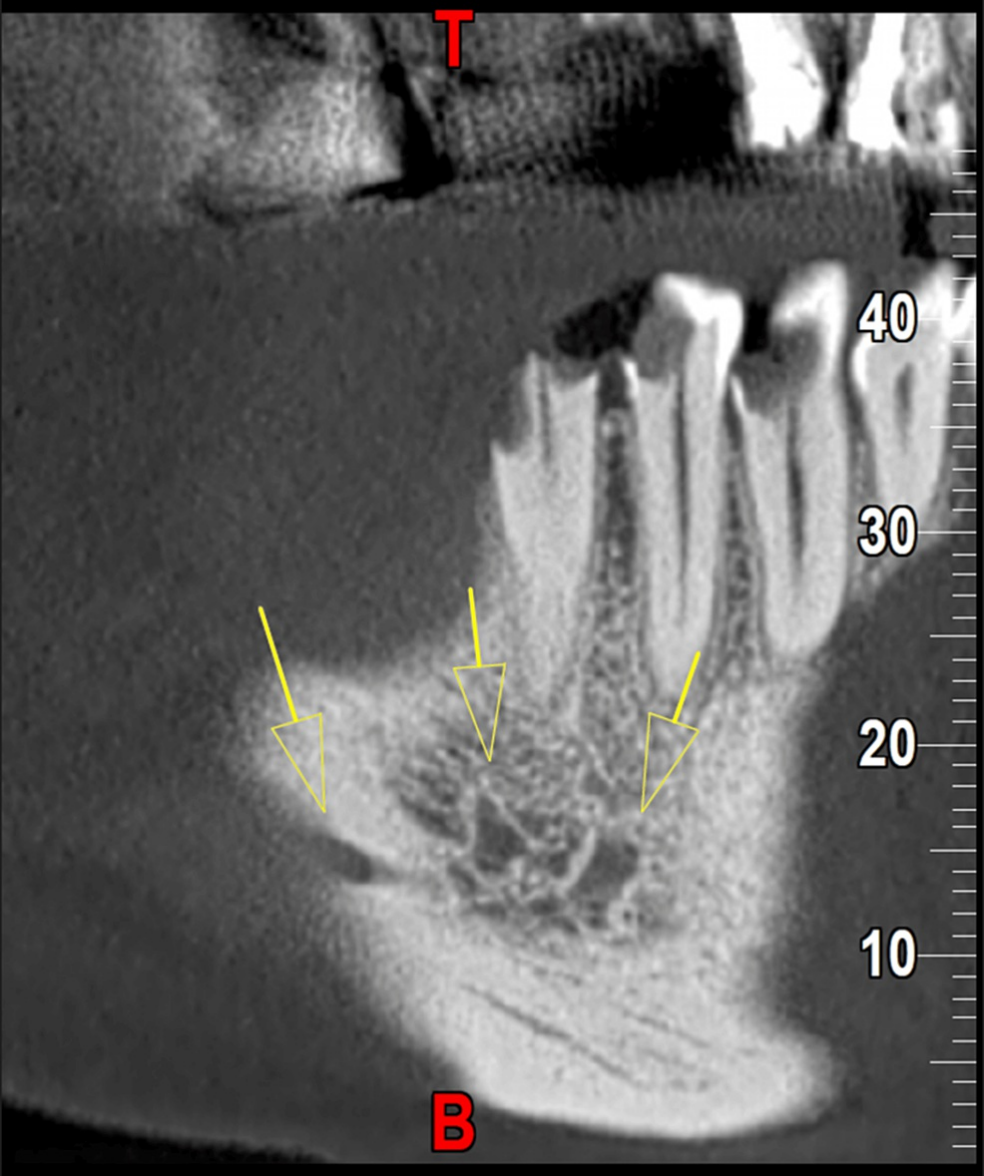
Sagittal view showing triple mental foramina

**Figure 3:**
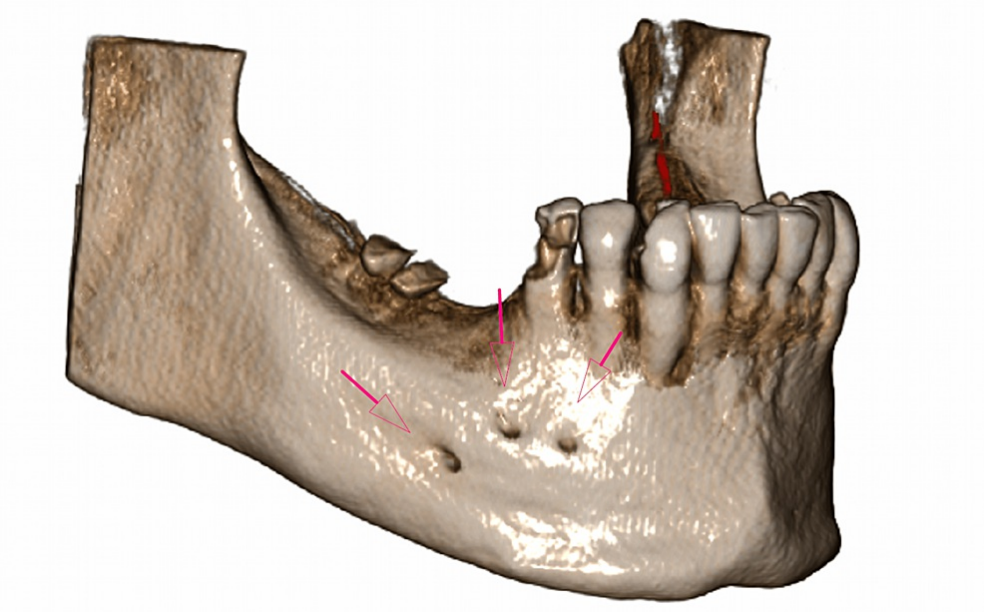
3D reconstruction from a CBCT volume showing three mental foramina in the buccal cortical plate CBCT: cone-beam computed tomography

**Figure 4:**
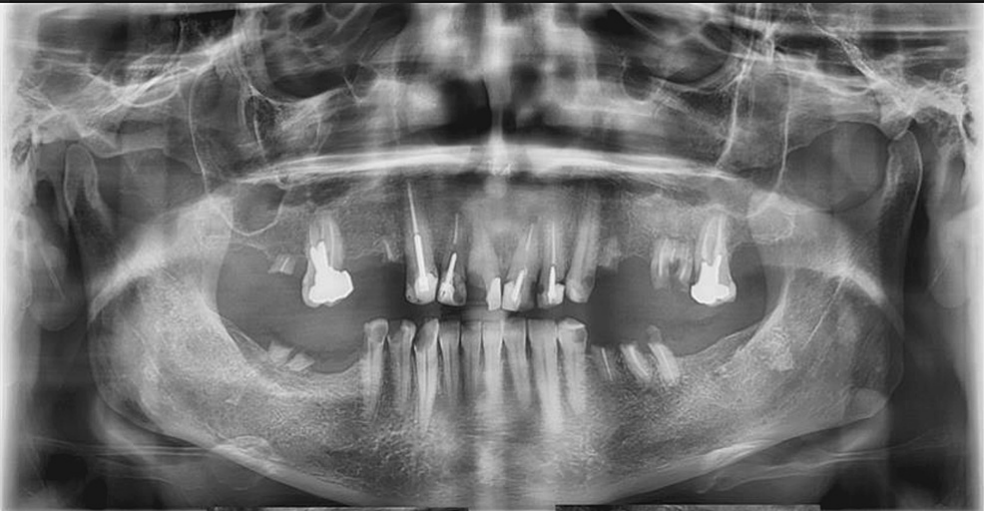
Panoramic view that doesn’t show the three mental foramina

The patient was a 51-year-old female whose dentist prescribed a CBCT of the posterior mandibular region of the right mandible for implant treatment. CBCT images were obtained using the New Tom CBCT imaging unit (Giano HR, Italy). The CBCT images were processed using NNT Viewer software (https://www.newtom.it/en/dentale/software/software-nnt/) and reconstructed to be viewed in cross-sectional and sagittal views (Figures 1, 2). In the evaluation of cross-sectional images, mental foramina were observed. After discovering the multiple mental foramina, reconstruction was done in the sagittal and axial planes. Three mental foramina were found in the buccal cortical plate (Figure 3), which could be seen in the distal and periapical region of the first premolar tooth, the distal and periapical region of the second premolar tooth, and the edentulous region of the first molar in the lower third region between the crest ridge and the lower cortex of the mandible. In the panoramic view, although the mental foramen and loop were clearly visible on the left side, none of them were visible on the right side where multiple mental foramina were observed in the CBCT (Figure 4).

Due to the rarity of this case, which presented an unusual normal anatomical variant, no specific treatment was given to the patient. The primary objective of this case report is to raise dentists’ awareness of the possible existence of such variations to prevent any iatrogenic errors during implantation procedures. Consequently, implant placement in the mandibular premolar-molar region should be approached with greater caution and conservatism.

Although clinicians should be more careful and conservative in examining these areas, the patient was reassured that the triple mental foramina anatomy is a rare normal variation and does not require any treatment.

## Discussion

The presence of accessory mental foramina has been mentioned in various studies. Whenever surgical treatment is planned in the area, a careful evaluation of the mental foramen is necessary to avoid possible injuries. The most common radiograph used to evaluate the mandible is the panoramic view, which, unfortunately, has low accuracy in determining the presence or exact position of the mental foramen. In this case report, accessory mental foramina were not visible in the panoramic view (Figure 4). In this paper, the significance of pre-implant treatment planning radiography and its crucial role in accurate anatomical identification and comprehension is emphasized. In this case, the mental foramina and their loops were not clearly visible in the initial panoramic radiograph but were successfully localized and detected using advanced modalities like CBCT.

The mental foramen is usually located in the area of ​​the apex of the second premolar and vertically almost in the middle of the distance between the lower ridge of the mandible and the crest of the alveolar ridge. The prevalence of accessory mental foramina is less than 10% [5], and in 90% of cases, it is unilateral and usually more common on the right side [6].

Less than half of the secondary mental foramina identified by CBCT are also seen in panoramic views [7]. In addition, the angle of the foramen might be oblique, which may not be identifiable [8].

The present case report includes a patient with two accessory mental foramina in addition to the main mental foramen on the right side of the mandible. The patient was referred for a CBCT scan of the implant placement area, and the presence of three mental foramina was randomly determined, with an impact on his implant treatment plan since one of the accessory mental foramina was more distal than the usual location of the mental foramen and was located in the area of ​​the first molar, which was a candidate for implant placement in this patient and required special attention in terms of neural structures.

According to past reports, accessory mental foramina are usually present in the apical region of the first molar and the posterior or lower region of the mental foramen. Also, the largest foramen is usually considered the mental foramen, and the distance between the mental foramen and the accessory foramen has been reported to be about 6 mm [9,10]. In this case, the dimensions of each of the foramen observed on the right side from mesial to distal were 0.5, 0.8, and 1.4 mm, the largest of which was in the distal, and the approximate distance between the most mesial foramen and the most distal foramen was about 5 mm (Figure 1). The reason for this difference might be the rarity of this case because most of the reports are about cases with one accessory foramina, but this case had three foramina, which, according to the available literature, cannot be accurately determined as the main foramen. In a study conducted by Watanabe on 100 dried human mandibles, although the prevalence of an accessory mental foramen was close to 40% in both sexes, triple foramina were not observed in any of the mandibles [11].

The reported cases of triple or multiple mental foramina in the literature comprise four or five reports. However, since the difference in accuracy in identifying the mental foramen between CBCT and panoramic radiography is significant, CBCT is more accurate and reliable. It is recommended that CBCT be used as a preoperative assessment tool to minimize surgical complications related to nerve damage during implant placement in the mandibular premolar and first molar region [12].

## Conclusions

Three-dimensional evaluation of the bone before placing implants is necessary to prepare the best treatment plan. Advanced imaging techniques, such as CBCT, are recommended due to their low dose and high resolution to accurately understand the anatomy and morphology of the mental foramen before invasive surgery, evaluate the condition of the remaining ridge in terms of width and height, and detect anatomical variations and possible pathology. These anatomical variations are often ignored, and dentists should be aware of these normal variations and avoid iatrogenic injuries during surgery.
